# Supplementary material for: Are carbon emissions trading and green financial instruments synergistic? -Comprehensive quantitative research based on content analysis
Source: PLoS One. 2024 Mar 7;19(3):e0298601. doi: 10.1371/journal.pone.0298601 (PMC10919601; doi:10.1371/journal.pone.0298601)
Supplement: S1 Data — (ZIP) [file pone.0298601.s001.zip › Data/地级市Z_F工作报告.zip!/╕ú╜¿/╧├├┼╩╨/└┤╫╘╣1⁄2╓┌║┼í╛┬φ┐╦╩2╛▌═°í┐_2008.docx]

各位代表：

　　现在，我代表市人民政府向大会作政府工作报告，请予审议，并请市政协委员和各位列席人员提出意见。

　　一、2007年的工作回顾

　　2007年是本届政府的开局之年。我们在中共厦门市委的领导下，以邓小平理论和“三个代表”重要思想为指导，全面落实科学发展观，努力构建社会主义和谐社会，积极融入海峡西岸经济区建设，持续推进新一轮跨越式发展，又好又快地完成了市十三届人大一次会议确定的主要任务。全年实现地区生产总值1375亿元，增长16.1%；工业总产值2837亿元，增长17.7%；全社会固定资产投资927亿元，增长40.1%；财政总收入348.4亿元，增长30.3%，其中地方级财政收入186.5亿元，增长37%；城镇居民人均可支配收入21503元，农民人均纯收入7637元，分别增长16.2%和11.2%；城镇登记失业率3.49%；居民消费价格指数104.6；人口出生率10.8‰；单位生产总值能耗等节能减排目标预计可以完成。

　　这一年，党的十七大胜利召开，全市人民备受鼓舞，奋力拼搏，开拓创新，增强了推动工作、推动建设、推动发展的强大合力，经济社会持续了增长较快、结构优化、效益提高、民生改善的良好态势。常住人口人均生产总值达到7398美元，财政收入比2004年翻了一番，各区经济实力显著增强，“四个加强”、“四个破解”取得明显成效，规模浩大的环东海域整治顺利推进，湖边水库、杏林湾等重大片区开发快速推进，城市面貌正在发生显著变化，社会保障性住房经验得到国家有关部门充分肯定，成功举办第十一届中国国际投资贸易洽谈会、第六届中国国际园林花卉博览会，厦门国际马拉松成为国际田联路跑金牌赛事，国际海洋周和中国国际钢琴比赛永久落户厦门，厦门位列中国城市和谐发展指数第二名，荣膺“全国双拥模范城市”六连冠、“全国绿化模范城市”和“跨国公司最佳投资城市”。

　　一年来，我们主要做了以下工作：

　　（一）着力调整经济结构，产业素质明显提升

　　三次产业结构由1.6∶53.9∶44.5调整为1.3∶53.5∶45.2，第三产业比重提高0.7个百分点，高新技术产业稳步发展，产业科技含量提高，经济发展方式进一步转变。

　　先进制造业进一步壮大。启动环东海域轻工电子工业集中区建设，火炬（翔安）产业区、同安工业集中区、集美机械工业集中区、出口加工区标准厂房和配套设施建设加快，厦华工业园、汽车工业城一期等产业集群项目进展顺利，火炬高新区成为国家科技兴贸创新基地和全国惟一的光电显示产业集群试点，金龙客车和金旅客车获得国家汽车整车出口资质。友达光电、柯达图文影像、龙欣利照明（T5节能灯）等项目竣工投产，法拉电子、星鲨药业搬迁进展顺利，晶宇光电、卓越能源等项目相继开工。重点扶持一批科技公共服务平台，钨材料工程技术研究中心成为钨产业国家核心研发平台，新增厦门汽车、通士达两家国家级企业技术中心，全市发明专利授权量增长31.5%，成为国家知识产权示范城市创建市。新认定111家高新技术企业，新增6个中国名牌产品、4个中国驰名商标称号，工业企业综合效益指数提高7.86个百分点。

　　现代服务业发展加快。引进中远集团、法国达飞集团等战略合作者落户海沧港区，马士基第六代集装箱船舶安全靠泊厦门港。开通厦门至南昌、赣州的集装箱海铁联运班列。东渡国际邮轮中心、五通海空联运码头竣工验收。开通厦门至阿姆斯特丹的航空货运航线。鼓浪屿被评为首批国家5A级旅游景区。鼓浪湾、翠丰温泉、福佑戴斯等高星级酒店投入营业。全年接待境内外游客突破2000万人次，其中境外游客超过110万人次。引进奥地利中央合作银行，厦门国际银行在北京设立分行，生命人寿、平安养老等五家保险公司落户厦门。思科、甲骨文、雅马哈信息等全球知名企业落户软件园。瑞景、加州商业广场投入使用，“农改超”稳步推进，北京佳龙等24家企业在厦设立营运中心。成功举办中国经济发展论坛、国际龙奖年会、中国建设银行全球董事会等重要会展活动，全年举办会议展览和大型活动超过1500场次，服务业在全市经济发展中的地位进一步提升。

　　（二）加快中心城市建设，基础设施日趋完善

　　环东海域、五缘湾、观音山等重大片区开发建设进展顺利，集美、海沧、同安、翔安新城区进一步拓展，会展北部城市综合体初见雏形。福厦、龙厦、厦深高速铁路建设顺利，杏林大桥、集美大桥、翔安隧道、快速公交系统（BRT）一期等加快推进，成功大道（北段）、同安大桥建成通车，仙岳路高架等一批交通改善工程投入使用，县黄路等7段“断头路”连接贯通。嵩屿港区一期工程、海沧港区7＃、9＃泊位建成并投入使用，招银、石码港区公共基础设施进一步完善，完成东渡航道等扩建工程。东部供水工程建设加快，完成同安梅山水厂扩建、北溪引水左干渠综合整治一期工程，海沧500KV输变电工程投入运行，完成进岛第二电力通道增容改造工程，环卫综合处理厂基本建成。城市综合保障能力不断提升，海峡西岸重要中心城市辐射带动作用进一步增强。

　　（三）加大“三农”工作力度，新农村建设取得新进展

　　大力发展“两头在厦、中间在外”的新型农业，订单农业基地达16万亩，带动农户12万户，全市34家市级农业产业化龙头企业实现加工产值140亿元，增长14%，银鹭、中盛等4家企业荣获省“品牌农业金奖企业”称号。实施“金包银”工程19个，新增股份合作经济项目8个，农民人均纯收入增幅连续3年超过10%。25个村实施旧村改造与新村建设，集美许庄村、翔安大帽山农场首批移民造福工程顺利推进。广泛开展农村“家园清洁行动”，完成生态风景林建设1.2万亩，自来水、有线电视入户率继续提高，农村道路硬化、绿化美化、环境整治和文明建设卓有成效。在全国率先实行被征地农民、上岸渔民与城镇职工同等享受基本养老保险制度，对实行“村改居”且失去农村集体土地承包权的居民及时按城镇居民标准享受待遇，农村新型合作医疗参保率达98.3%。采取政府购买公益性岗位和社保补助的办法，帮助上岸渔民和大龄农民转移就业。全年完成农村劳动力转移培训7625人、实现就业24415人。继续挂钩帮扶经济薄弱村，政府资助农村贫困家庭子女学费，村干部基本报酬财政统筹制度逐步建立。

　　（四）致力体制机制创新，改革开放取得新成绩

　　深化国有企业改革，培养壮大了一批国有龙头企业。出台推进企业上市的意见，支持企业改制上市融资和上市公司再融资，东南融通成为在纽约证交所成功上市的首家中国软件企业，建发股份等四家上市公司实现再融资32.5亿元。完善重点建设片区指挥部模式，适时将同安工业集中区、软件园产业基地等项目指挥部转为管委会，实施常规化管理。完善征地拆迁政策，市级财政性投融资项目拆迁补偿实行总额包干，实行工业用地挂牌出让制度。完善建设工程招投标机制，规范项目竣工联合验收制度。积极推进公房管理市场化运作。实行财政支出绩效评价，推行公务卡改革试点，深化行政资源和社会公共资源配置市场化改革。稳步推进事业单位分类改革，完成一批事业单位清理整顿。

　　积极适应国家出口退税、加工贸易政策调整，认真落实出口扶持政策，在全国率先实行入仓退税制度和出口退税核销无纸化制度，加快退税进度。支持企业到境外设立贸易网点以及参加各种商品展览会、交易会，成功举办第二届中国（厦门）品牌产品国际采购交易会。厦门港口岸管理一体化深入推进，漳州检验检疫局整建制划转厦门局管理，厦门港实现“关检合一”。加快电子口岸信息平台建设，率先实行关检通关单电子联网，推行“属地申报、口岸验放”通关模式，开通24小时通关服务热线，外贸进出口总额397.8亿美元，增长21.3%，其中出口255.5亿美元，增长24.6%。招商引资创历史新高，合同利用外资32.7亿美元，增长41.6%，实际利用外资12.7亿美元，增长33.2%；协议内资435.9亿元，增长13%，实际利用内资209亿元，增长29.8%，非公有制经济健康发展。促进对口支援与帮扶地区加快发展，进一步密切区域协作，厦泉漳龙城市联盟不断加强，闽粤赣十三市合作更加紧密。

　　（五）发挥对台五缘优势，两岸交流合作不断拓展

　　全年合同利用台资（含第三地）6.7亿美元，实际利用台资6.23亿美元。台湾人寿保险落户厦门，TDK、正新轮胎在厦设立研发中心，台湾最大种苗公司农友种苗在厦设立总部，思源科技、泰伟电子等知名台湾软件企业落户厦门软件园。长庚医院、翔鹭体检中心开始营业，奥林匹克网球中心、奥林匹克博物馆建成投入使用，台湾科技企业育成中心、闽台（厦门）花卉高科技园、对台花卉进出口市场等项目进展顺利。启动福建居民经厦门至澎湖旅游，厦金旅游成倍增长，厦金航线全年运送旅客67.7万人次，开通至澎湖货运直航，集装箱从台湾转运到厦门中转。厦门口岸台湾水果年进口量占全国三分之一。成功举办第十一届“台交会”、首届海峡两岸动漫展、海峡两岸民间艺术节、第二届两岸青年联欢节、海峡两岸农产品产销论坛、第二届保生慈济文化节、第二届海峡两岸中医药发展与合作论坛、第三届海峡旅游博览会、第三届海峡两岸图书交易会，赴台举办海峡两岸建筑建材展，厦门卫视实现对亚洲、欧洲和美国的覆盖。对台湾学生实行同等收费，设立奖助基金。小白鹭民间舞团、爱乐乐团和翔安民间戏曲学校赴台演出，组织郑成功文物赴台展览，厦门与台湾的经贸文化交流更加密切。

　　（六）落实“四个加强”、“四个破解”，和谐社会建设取得明显成效

　　全市财政用于教育、卫生、文化、体育等社会事业支出41亿元，增长24.3%。在全省率先实现“双高普九”，率先实现城乡义务教育阶段免收学杂费。厦门软件职业技术学院、厦门医高专、高级技工学校新校区建成投入使用，厦门理工学院二期、华侨大学集美校区等项目顺利推进，开工建设厦门二中高中部、五缘湾实验学校、中央音乐学院鼓浪屿钢琴学校。启动岛内38个义务教育建设项目。建立民办学校预警和退出机制。全面完成公共卫生体系建设，第一医院、中山医院被评为三级甲等综合医院，第二医院、第三医院被评为三级乙等综合医院，全市新增医院床位1500张，市民健康信息系统投入试运行。加强人口计生工作，出生人口政策符合率97.4%。加强文化遗产保护，国家授牌设点闽南文化生态保护实验区。文化艺术中心全面投入使用，中华教育园、南音阁和海沧、翔安文化活动中心对外开放，小白鹭艺术中心建设进展顺利，歌仔戏《邵江海》和小说《天堂没有路标》获“五个一”工程奖，《邵江海》并获文华大奖。成功举办厦门国际马拉松赛和全省第六届农民运动会，在第六届全国城运会上取得优异成绩，国家乒乓球南方训练基地等项目进展顺利，全民健身运动广泛开展。

　　重视生态文明建设和环境保护工作，建立污染物减排监控体系，加强对工业废气、汽车尾气、工地噪声、道路扬尘、餐饮业油烟等污染综合治理。强化职业技能培训和创业培训，实施困难家庭毕业生就业社保补贴制度，全年新增就业19.8万人。社会保险覆盖面进一步扩大，在全国率先实施包括老年人、未成年人在内的全民医保，提高了养老金、失业保险金、工伤保险金、最低工资标准和低收入家庭困难补助，社保基金运行安全平稳。应对副食品价格上涨，对低保和低保边缘户4.3万人实行了临时补贴。55万农民工纳入医疗保险，建筑和矿山等行业的16万农民工纳入工伤保险。推进工业集中区周边的综合服务中心和外口公寓建设，采取有效措施降低农民工房租水电费支出，改善农民工生活环境。开工建设集美滨水小区等13个社会保障性住房项目，1600多个家庭选到自己满意的住房。加大中心城区交通环境综合整治力度，努力缓解交通拥堵问题。加快推进全市突发公共事件应急管理体系建设，健全防汛防台风工作机制。产品质量和食品药品安全专项整治通过国家验收。加强安全生产专项整治，较好完成安全生产责任控制目标。推进“平安厦门”建设，依法严厉打击严重刑事犯罪，群众对社会治安满意率得到巩固。加强信访工作，完善多元化纠纷解决机制，积极化解社会矛盾。对经济社会发展中群众关注的重大事项，认真听取意见，积极引导公民依法有序理性表达诉求，既促进问题的解决，又维护了社会和谐稳定。广泛开展“迎奥运、讲文明、树新风、促和谐”活动，加快和谐社区、和谐村镇、和谐行业建设，文明城市创建成果巩固提升。十件为民办实事项目基本完成。外事、侨务、人事、编制、气象、防震、方志、档案、信息、安全等工作得到加强。双拥共建、人防海防、民兵预备役、民族宗教、妇女儿童、老龄、残疾人等工作取得新成绩。

　　（七）加强民主法制建设，依法行政水平不断提高

　　全面推进依法行政，修订和完善政府工作规则，制定和修订11件政府规章，聘任市政府法律顾问，建立市政府部门规范性文件公布前统一审查制度，积极探索行政复议调解结案方式，开展规范行政自由裁量权试点工作。完成公务员登记工作。全面清理行政审批项目，实现网上受理和预审行政审批项目214项，备案和年检年审事项基本实现网上办理。加大行政效能督查和纠风工作力度，推行“一站式”服务模式，群众对服务窗口的满意率进一步提高。落实廉洁自律各项规定，惩治和预防腐败体系逐步完善。加强审计监察工作，专项治理商业贿赂工作取得成效。坚持向人大及其常委会报告工作，依法接受人大法律监督和工作监督，自觉接受政协民主监督，加强同民主党派、工商联和社会团体的联系。认真办理人大代表议案33件、建议233件，政协提案574件，办结率100%，满意率分别达95.6%和97.9%。

　　各位代表！

　　以上成绩的取得，是在中共中央、国务院和中共福建省委、省政府及中共厦门市委的正确领导下，在各级人大及各位代表的依法监督和有力支持下，在各级政协及各位委员的民主监督和积极参与下，全市干部群众齐心协力、艰苦奋斗的结果。在此，我谨代表市人民政府，向全市人民，向各民主党派和工商联、人民团体、各界爱国人士，向离退休的老同志，向中央和省驻厦单位，向驻厦人民解放军、武警官兵和公安干警，向来厦的所有投资者和外来劳动者，向所有关心支持厦门的港澳同胞、台湾同胞、海外侨胞和国际友人，表示衷心的感谢和崇高的敬意！

　　一年的政府工作给我们留下深刻体会：一是必须坚持以科学发展观为统领，统筹兼顾各项事业发展，把贯彻落实科学发展观体现在经济社会发展的各个领域、各个方面。二是必须坚持改革开放，不断增创经济特区发展新优势，勇于冲破妨碍发展的思想观念，勇于改变束缚发展的做法和规定，勇于革除制约发展的体制机制，积极参与国际经济技术合作和竞争，为经济特区发展不断注入新的活力。三是必须坚持把党中央国务院、省委省政府的决策部署与厦门实际紧密结合，创造性地开展工作，找准工作的切入点，不断探索创新推进工作的策略与方法，提高驾驭经济社会发展的能力和水平。四是必须坚持调动一切积极因素，把各方面的积极性保护好、引导好、发挥好，持久形成推进工作的强大合力，持久形成推进新一轮跨越式发展的氛围和气势，持久形成风正气顺、人和业兴的良好局面。

　　各位代表，我们也清醒地看到，我市经济社会发展还面临一些亟待解决的困难和问题，突出表现在：一是经济转型和产业结构战略性调整的任务依然艰巨，产业链中高端项目和产业规模大的项目不够多，现代服务业的发展还需进一步加快；二是征地拆迁难度仍然较大，一些重点工程的推进受到影响；三是自主创新能力不足，创新型人才和高技能人才短缺；四是农民转产转业和增收难度加大，大部分“村改居”社区仍未改变原来的农村管理模式；五是物价、房价涨幅虽然低于全国全省平均水平，但抑制物价、房价任务仍然艰巨；六是改善空气质量和节能减排任务较为艰巨，交通拥堵现象仍然存在；七是城市管理、社会管理的实际成效与群众的期望和要求还有较大差距；八是政风行风建设中办事效率不高、行政不作为的现象仍然存在，少数干部在困难和问题面前存在畏难情绪，应对形势变化和政策调整的能力不强，预防和处置突发事件还需更加主动有效。对这些问题，我们要高度重视，并将在今后工作中切实加以解决。

　　二、2008年主要工作

　　2008年，是改革开放三十周年，也是我市完成“十一五”规划的关键一年。一方面，党的十七大明确提出积极支持东部地区率先发展、更好地发挥经济特区在改革开放和自主创新中的重要作用、支持海峡西岸和其他台商投资相对集中地区经济发展，为我们推进新一轮跨越式发展提供了重要的历史机遇。另一方面，国际国内经济形势错综复杂，经济发展中的不确定因素增多，国家实施稳健的财政政策和从紧的货币政策，宏观调控力度将进一步加大，内外资企业所得税“两税合一”、人民币汇率变动、国际原油价格持续攀升、原材料和劳动力成本上升，地区产业梯度正加速形成。面对新形势、新情况，我们要认真总结改革开放三十年来的成功经验，积极探索新时期经济特区发展规律，进一步解放思想，扩大开放，以改革创新精神推进经济社会又好又快发展。

　　2008年政府工作的总体要求是：全面贯彻党的十七大精神，高举中国特色社会主义伟大旗帜，以邓小平理论和“三个代表”重要思想为指导，深入贯彻落实科学发展观，积极构建社会主义和谐社会，按照省第八次党代会精神和建设海峡西岸经济区的总体部署，认真落实市第十次党代会精神，把“四谋发展”实践主题、“四个重在”实践要领、“四个关键”实践要求、“四求先行”实践方向贯穿于各项工作中，实施项目带动和品牌带动，着力推进改革开放和自主创新，着力优化经济结构和提高经济发展质量，更加重视节能减排和生态环境保护，更加重视改善民生和促进社会和谐，又好又快地扎实推进新一轮跨越式发展，努力在海峡西岸建设科学发展的先行区、两岸人民交流合作的先行区中作示范当榜样，努力为推进海峡西岸经济区建设和促进祖国统一大业做出历史性贡献。

　　2008年主要预期目标是：地区生产总值增长15%，地方级财政收入增长23%，城镇居民人均可支配收入和农民人均纯收入均增长11%，城镇登记失业率控制在4%以内，人口出生率控制在11.4‰以内，居民消费价格指数略低于2007年实际涨幅。单位生产总值能耗降低2.52%，二氧化硫排放量下降1.1%，化学需氧量减少1.3%。

　　实现上述目标，我们必须扎实做好以下工作：

　　（一）转变经济发展方式，推动产业结构优化升级

　　着力发展先进制造业。强化产业规划和政策导向，制定鼓励产业配套的政策，推进信息化与工业化融合，推动产业向高端化发展。大力培育高新技术企业，着力推动航空维修、光电显示、汽车电子、电工电气、生物与新医药等产业发展，做强做大产业链。抓紧完善工业集中区的基础设施配套和服务设施配套，推进汽车工业城二期、节能光源制造基地建设，推动更多企业入驻、投产达产，力促工业园区早出效益。突出抓好工业新增长点项目，促进友达光电、宸鸿科技、达运精密等企业尽快达产，推动安台创新科技等一批项目竣工投产，开工建设新科宇航等一批项目。深入实施品牌带动战略，鼓励企业增加技改投入，注重用高新技术和先进适用技术改造提升传统产业，扶持发展拥有本土自主知识产权和自主品牌的项目，促进产品升级换代。

　　加快发展现代服务业。制定鼓励现代服务业发展的政策措施，重视发展与高端制造业相互融合的高端服务业。着力培育海峡西岸航运物流、旅游会展、商务营运、金融保险、文化教育、医疗卫生中心。推进现代物流园区建设，推进保税区、保税物流园区和东渡港区“三区合一”，争取设立海沧保税港区、火炬（翔安）B型保税物流中心。扶持码头企业开辟新货种，培植大宗商品货源，推动海铁联运，发展内陆“无水港”。加强与马士基、中远等国际知名航商合作，大力发展国际中转业务，鼓励发展第三方物流。加强旅游、商贸、会展、文化之间的互动，争创全国最佳商务会展旅游城市。加强城市旅游整体营销，推动旅游产业由观光型向商务会展、休闲度假型转变，做好海上邮轮、游艇和帆船文章，支持发展园林博览、康体温泉、夜间文化演出等休闲旅游产品。加快会展中心二期和国际会议中心建设，进一步办好“投洽会”、石材展等重大会展活动，争取更多的大型展会来厦举办。抓好特色商业街建设，引进高档商业购物中心。完善扶持金融业发展的政策措施，着力引进境内外金融机构，推动优质企业在境内外上市融资和上市公司再融资。积极引进知名软件企业，加快发展集成电路设计、动漫游戏、信息技术服务外包等产业。大力培育油画、动漫、钢琴、演艺和艺术品拍卖、图书发行等文化产业。积极引进境内外知名中介服务机构，加快发展法律、会计、评估、咨询等中介服务业。

　　强化节能减排和资源综合利用。认真落实节能减排工作责任制，严格固定资产投资项目节能评估审查制度，扎实开展第一次全国污染源普查，强化工业、建筑、交通运输、商业等重点领域的节能工作，有效减少污染物排放。推进循环经济试点，发展环保产业，推广节约、替代、循环利用和治理污染的先进适用技术，推广太阳能路灯、中水回用、海水综合利用等节能示范项目，创建全国节水型城市。综合利用土地资源，开发盘活存量土地，推行工业用地预申请制度，强化节约和集约用地，严格保护耕地。

　　（二）着力深化改革开放，激发经济特区发展活力

　　推进体制机制创新。深化国有企业公司制、股份制改革，加快建立国有资本经营预算制度，完善国有企业负责人经营业绩考核制度，健全现代企业制度。加强项目与资金对接，把财政资金、信贷投资、外商投资和社会民间资本结合起来，形成多元化投融资体制。整合重点工程建设管理机构，完善协调机制。加快推进事业单位分类改革，着力推进教育、医疗卫生和社会保障、收入分配等民生领域改革，稳步推进文化体制改革综合试点工作，进一步深化广电体制改革，增强社会事业发展活力。

　　提高招商引资质量。充分利用“投洽会”、“台交会”、“6·18”项目对接会等平台，做好工业集中区、软件园产业基地、营运中心区的招商引资，鼓励现有企业增资扩产，优化引资结构，提升引资质量。依托现有产业链和产业集群，引进带动力强的高科技、高附加值、节能环保型的高端制造业。积极引进科技研发、航运物流、现代金融、旅游会展、医疗卫生等服务业的高端部分，吸引跨国公司和国内外企业在厦设立研发中心、销售中心和地区总部，发展服务贸易，争取成为国家级服务外包基地。

　　扩大对内对外贸易。推进科技兴贸，加大力度扶持附加值高、科技含量高、有发展潜力的出口商品，扩大客车、钨制品、游艇等优势产品出口，培育电子、工业原辅料等商品的进口分拨中心，扩大信用通关，优化通关流程，提高口岸便利化程度，把厦门建设成为我国重要的进出口商品集散地。集中用好专项资金，支持企业拓展新的市场。继续办好中国（厦门）品牌产品国际采购交易会，扩大地产工业品的市场份额。鼓励有条件的企业稳步实施“走出去”战略。进一步推进区域协作，加强厦泉漳龙城市联盟，密切闽粤赣十三市的合作，认真做好对口支援、对口帮扶工作。

　　提升城市国际化水平。加强与菲律宾、新加坡和泰国驻厦领事机构的交往，加快领馆区建设，创造条件吸引更多国家来厦设立领事机构。加强与国际友城、国际友港的实质性交流，开展互利互惠合作。用足用活出国审批权限，简化人员出入境审批和口岸通关手续，完善各项配套服务，创建国际卫生海港、国际卫生机场，把厦门打造成东南沿海通关便捷、服务周到的出入境口岸。加快与国际接轨，不断提升国际化水平和厦门在国际上的知名度。

　　（三）提高自主创新能力，加快建设科技创新型城市

　　完善科技创新体系。持续增加科技投入，重点支持科技创新公共平台建设，扶持拥有自主知识产权的重大产业科技项目。引导创新要素向企业集中，鼓励企业、高校、科研机构建立创新联盟，扶持企业技术改造和设立研发中心，支持企业参与制定并采用国际标准、国家及行业标准。加快建设环东海域火炬东海科技园、中科院城市环境研究所，支持筹建厦门产业技术研究院、车辆与工程机械研发中心。注重保护知识产权，推动国家知识产权示范城市创建工作。加强科普教育，提高市民科学素质。

　　加强创新人才队伍建设。加快培养和引进一批具有自主创新能力的高层次人才、高技能人才。健全人才选拔任用机制和聘用、兼职等聚才引智机制，加强人才储备，促进人才与项目对接、与产业互动。强化职业技术教育和技能培训，推行校企“订单式”合作培养模式，加快高技能人才公共实训基地建设，培养与厦门产业发展相适应的高素质技能型人才。在社会保障、住房等方面创造条件，让人才引得来、留得住、用得好。进一步营造鼓励创新的环境，使全社会创新智慧竞相迸发、创新人才大量涌现。

　　（四）加强城市建设管理，提升中心城市综合承载能力

　　加快城市基础设施建设。集中资金保证重点工程和重大片区开发建设，加快推进五缘湾、观音山等商务营运中心建设和环东海域、杏林湾、湖边水库综合整治，全面开发厦门（新）站片区、前场铁路特大型货场。建成集美大桥、杏林大桥和成功大道，继续加快翔安隧道、快速公交系统（BRT）和海翔大道等项目建设，大力推进福厦、龙厦、厦深高速铁路及厦门—安溪城际快速路建设。继续推进海沧、浏五店等港区码头泊位建设，加快厦门港主航道、海沧航道、招银航道扩建工程。推进机场三期建设。加快建设官浔等一批输变电工程。加快LNG项目建设，完成“西水东调”工程，启动九龙江北溪引水改善工程，推进汀溪水库群综合整治和莲花水库等工程建设，扩建石胄头和同安污水厂，推进重大片区的防洪排涝设施建设。推进海沧、翔安垃圾焚烧发电厂建设，建成东部固废处理中心垃圾填埋场。

　　提高城市管理水平。加强城市空间规划、利用与管理，做好重点地区、重点地段的城市设计和建筑精品设计，精心打造城市亮点。保护好历史文化风貌区和优秀历史建筑，传承城市历史文脉。完善市民、法人、地理空间基础数据库，推进信息资源共享和运用。开展数字城管信息平台建设，逐步推进数字化城市管理，完善城市管理长效机制。深入治理汽车尾气、工业废气、扬尘污染、油烟噪声扰民，强化对违法建设的执法力度，加强LED夜景工程维护管理。大力推进市容环境综合管理，进一步改善市容市貌。

　　持续改善人居环境。全面推进生态文明建设，续建园博园、植物园和忠仑公园，启动狐尾山气象主题公园建设，推进莲花、大帽山森林公园建设，增加绿地700公顷，实施生态风景林改造1.2万亩，推进绿色通道工程建设，把国道、高速公路建成生态景观道路。建立环境应急预警系统，加强重点污染源环境安全监管，严防各类污染事件发生。通过区域合作减少九龙江入海污染物。实施海洋生态修复，推进海堤改造、人造沙滩、红树林种植和湿地公园建设。

　　（五）发挥前沿平台作用，建设两岸交流合作先行区

　　促进两岸产业对接。继续办好海沧、杏林、集美三个台商投资区，积极承接台湾产业转移，争取在光电、软件、动漫产业和服务外包等方面取得更大突破。鼓励台资企业在厦设立总部，加大力度引进台湾航运物流、现代金融、医疗教育和中介服务业。继续支持长庚医院建设，引进更多的先进医疗技术和管理经验。深化对台农业合作，扩大台湾新品种的引进示范，加快建设闽台（厦门）花卉高科技园、闽台中心渔港等对台农业合作项目。

　　推进对台经贸合作。做大做强“台交会”、“两岸图书交易会”、“食博会”等涉台展会，积极组织赴台办展参展。以台湾水果销售集散中心为平台，落实扶持政策，做大台湾农产品进口规模，改扩建大嶝对台小额商品市场。改善厦金直航通关条件，继续做大厦金小三通，拓展厦金航线包裹业务和客船带运小件货物运输业务，继续做好对金门蔬菜、大米出口。

　　密切两岸人员往来和文化交流。积极争取扩大赴金门、澎湖旅游客源范围，发挥好厦门口岸在福建居民赴澎湖旅游的枢纽作用，努力争取成为首批大陆居民赴台旅游口岸。继续推动两岸客运直航包机周末化。利用闽南文化的特色和优势，全方位、多层次开展与台湾各界、各阶层的交往，深化科技、教育、文艺等各领域的交流。办好海峡两岸文化创意产业博览交易会、海峡两岸艺术节、第三届保生慈济文化节和“第三届世界金门日”。深化厦门与台中市的交流合作。进一步办好厦门卫视、闽南之声广播、厦门网。

　　（六）统筹协调城乡发展，加快社会主义新农村建设

　　推进农业现代化。按照“两头在厦、中间在外”的都市型现代农业发展模式，大力发展优良种苗业、高附加值加工销售业以及生态休闲农业。加大对农业产业化龙头企业的扶持力度，拓展订单农业，支持企业到外地建立基地和农产品销售网络。推动种苗业向工厂化生产、产业化经营的方向发展，将厦门建成种苗研发、繁育和供应的集散地。扩大无公害农产品生产，提高农产品安全质量及市场竞争力。开发乡村旅游资源，培育一批“农家乐”示范点。

　　加快农村城市化。通过工业集中区、重大片区、重大产业项目带动农村城市化。做好“镇改街、村改居”后续工作，加大对社区的公共投入，开展农村社区建设试点工作，扩大农村公共服务范围。新启动23个重点村进行旧村改造和新村建设，推进边远山区移民造福工程。加快农村自来水、有线电视进村入户，推进村道硬质化向自然村延伸，增辟农村客运线路，加快农村客运站点和交通安全设施建设，完善农村信息服务平台。启动新一轮农村义务教育、公共卫生体系建设。深入开展农村“家园清洁”行动，建设一批农村环卫设施，完善农村垃圾清运处理体系。续建九溪、过芸溪、瑶山溪等流域综合治理工程，实施水库堤坝新一轮除险加固，增强农村防洪排涝能力。

　　提高农民生活水平。加快新建一批“金包银”工程，发展农民股份合作经济，抓好项目招商招租和股份配售。大力开展职业技能培训，将被征地农民和上岸渔民纳入再就业政策扶持范围，为大龄农民提供公益性和服务性岗位，力争完成农村劳动力转移就业1.5万人。全面落实被征地人员基本养老保险制度，完善农村居民基本医疗保险制度，进一步加大政府补助力度，调整农民参保的统筹标准，将门诊常见病、多发病纳入保障范围，实现城乡居民基本医疗保险制度的统一。调整农村最低生活保障标准，做到应保尽保。积极推动老区山区村建设，加快致富奔小康步伐。

　　（七）深入推进“四个加强”、“四个破解”，积极构建和谐社会

　　坚持教育优先发展。促进城乡义务教育均衡发展，推动优质教育资源向农村辐射。重视学前教育，加强素质教育，减轻中小学生过重课业负担，积极稳妥推进高中课改。在新城区、工业集中区新建扩建一批中小学校，扩大农民工子女在公办学校就读机会。推进集美文教区、翔安文教区建设，继续共建厦大、集大，支持厦大翔安新校区建设，完成五缘学村等项目建设。加强教师队伍建设，提高教育教学质量。大力发展职业教育、华文教育、远程教育和继续教育，建设全民学习、终身学习的学习型社会。

　　提高医疗卫生水平。坚持公共医疗卫生的公益性质，建设覆盖城乡居民的公共卫生服务体系、医疗服务体系、医疗保障体系、药品供应保障体系。继续推进五缘医院等一批医院新建扩建项目建设，做好翔安医院、儿童医院等新建项目的前期工作，支持中医院创建三级甲等中医院。加强医德医风建设，开展惠民医疗服务，全面推广市民健康信息系统，提高医疗服务质量。强化药品安全监管，保障用药安全。普及医疗急救知识，不断完善公共卫生应急体系，建设全国一流的医疗急救体系。加强农村爱国卫生工作，建立覆盖全市农村的卫生监测网。继续稳定低生育水平，做好“三优”工作，抓好出生人口性别比偏高的治理，积极应对人口老龄化。

　　着力保障和改善民生。认真贯彻劳动合同法和就业促进法，完善面向困难群众的就业援助制度，推进残疾人按比例就业，开展订单式劳动力培训，将创业培训延伸到农村居民和城镇新增劳动力，力争全年新增就业20万人。落实职工带薪休假制度，提高最低工资标准，全面推行工资支付监控和工资保证金制度，建立健全解决拖欠农民工工资的长效机制，构建和谐劳动关系。扩大社会保险覆盖面，降低医疗保险门诊医疗费个人自付比例，继续提高企业离退休人员养老金和城乡低保补助标准，完善外来务工人员工伤保险制度，加强社会保险基金的安全监管，健全城乡一体的多层次的社会保障体系。促进房地产业健康稳定发展，新开工沧林花园等社会保障性住房项目，重点推动莲花六村、嘉福花园等35个安置房项目建设。加强交通组织管理，积极发展智能交通，新建一批人行天桥、地下通道和公共停车场，推行公交票制改革，提高公交服务质量。做好粮油肉储备，确保农副产品的供应，加强城乡市场监管，保持物价基本稳定。深化产品质量和食品药品安全专项整治，强化以粮食、蔬菜、水产品、肉品为重点的食品检验检测，让市民吃上放心食品。完善社会救助体系，发展慈善事业和社会福利事业，加快爱心护理院建设，抓好市福利中心扩改建工程，重点做好扶老、救孤、助残、济困工作。

　　切实维护社会安全稳定。围绕构筑“平安厦门”，全面落实社会治安综合治理责任制，改革和加强城乡社区警务工作，健全电子监控网络，合理配置一线警力，提高路面见警率和管事率，完善社会治安防控体系，依法严厉打击各类违法犯罪活动，保障人民生命财产安全。做好信访工作，进一步完善适应社会转型的利益协调、诉求表达、矛盾调处和权益保障机制。完善应急管理体制机制和预案体系，推进应急指挥平台建设，提高防灾减灾和处置突发事件的能力，保障公共安全。落实安全生产监管责任制，严格安全生产许可制度，建立健全重大危险源安全监控体系，重点做好海陆交通、危险化学品、非煤矿山、建筑施工安全专项整治，及时消除各类事故隐患，努力营造安全的社会环境。

　　（八）推动文化大发展大繁荣，持续创建全国文明城市

　　繁荣发展文化事业。办好鼓浪屿钢琴节暨全国青少年钢琴比赛等赛事活动。积极推动闽南戏曲艺术剧院建设，建成小白鹭艺术中心、嘉庚纪念馆、鼓浪屿乐器博物馆和风琴博物馆以及一批区级文化基础设施项目。认真做好第三次全国文物普查，推动鼓浪屿、闽南童谣等申报国家级非物质文化遗产，加强闽南文化生态保护。繁荣发展哲学社会科学，积极发展新闻出版、广播影视、文学艺术事业，加强档案保护，续修《厦门市志》。着力丰富农村、偏远地区居民和进城务工人员的精神文化生活，开展“书香鹭岛”活动月，推进“农家书屋”工程建设，营造阅读氛围，鼓励好书交换，让更多市民分享阅读的乐趣。建成工人体育馆、翔安体育中心、国家乒乓球训练基地，组织好2008北京奥运会火炬传递活动，继续办好厦门国际马拉松赛，以“全民健身与奥运同行”为主题，广泛开展群众体育活动。

　　做好新一轮全国文明城市创建工作。深入开展文明社区、文明村镇、文明行业等群众性文明创建活动。贯彻落实《公民道德建设实施纲要》，以社会主义核心价值体系为引领，弘扬和践行社会主义荣辱观。加强未成年人思想道德建设和大学生思想政治工作，净化网络文化环境。更加关心进城务工人员，在150个社区建立流动人口服务管理站。加强普法教育，增强全社会法制观念，推进社会诚信体系建设。充分发挥工会、共青团、妇联等群团组织以及行业协会作用，切实做好民族宗教、外事侨务工作。加强人防海防建设，完善国防动员机制，关心支持驻厦部队建设，积极构建优抚安置工作新机制，深化双拥共建活动。

　　三、继续推进政府管理创新，努力建设服务型政府

　　始终坚持把实现好、维护好、发展好人民的根本利益作为一切工作的出发点和落脚点，全心全意为人民服务，努力做到在服务中创新管理，在管理中体现服务。

　　增强发展能力，推进为民服务。各级政府领导和工作人员要用世界的眼光、战略的思维谋求发展，加强宏观经济运行分析，及时研究政策，善于从国内外形势发展变化中把握发展机遇、应对风险挑战，善于从新一轮跨越式发展实践中创新发展方式、破解发展难题，着力解决影响和制约科学发展的突出问题，着力解决人民群众最关心、最直接、最现实的利益问题，增强贯彻落实科学发展观的自觉性和坚定性，把以人为本贯穿于发展的全过程，做到发展为了人民、发展依靠人民、发展成果全民共享。

　　加快转变职能，完善为民服务。按照权责一致、分工合理、决策科学、执行顺畅、监督有力的要求，深化行政管理体制改革。完善政府社会管理和公共服务职能，把政府工作重点、政策支持重点、财力保障重点逐步向社会事业、公共事业发展倾斜，向基础设施和公共设施建设倾斜，向生态建设和环境保护倾斜，向扩大就业、完善社会保障体系、改善群众生活倾斜，向保障公共安全、维护社会和谐稳定倾斜。合理配置行政资产，加大资源整合力度，探索实行职能有机统一的运行体制，完善市区财政管理体制和社会管理方式。深入推进政企分开、政资分开、政事分开、政府与市场中介组织分开，把政府抓经济的主要精力放到为各类市场主体服务和创造良好发展环境上来。推进电子政务、网上审批。减少和规范行政审批，健全科学的政府绩效评估体系和经济社会发展综合评价体系，做好第二次全国经济普查工作，搞好政务督查，不断提高政府服务效率。

　　坚持依法行政，强化为民服务。贯彻落实《监督法》、《全面推进依法行政实施纲要》和《政府信息公开条例》，严格依照法定权限和程序行使权力、履行职责。加强行政立法工作，进一步完善行政执法体制，扩大规范行政自由裁量权试点。做好行政执法评议考核、过错责任追究和行政赔偿工作，加强基层复议机构建设，完善行政复议制度。健全重大事项专家咨询、公众参与、集体决策制度。依法接受市人大及其常委会的监督，自觉接受市政协的民主监督，坚持定期向市人大及其常委会报告工作和向市政协通报情况。认真听取民主党派、工商联、无党派人士和各人民团体的意见。认真做好人大代表议案、建议和政协提案的办理工作，重视新闻舆论和社会公众监督，支持监察、审计部门依法独立监督。依法深入推进政府信息公开，完善政府公报、政府网站、政府新闻发布会、政府信息查阅场所等公开渠道，保障人民的知情权、参与权、表达权、监督权。

　　不断改进作风，倾心为民服务。始终坚持群众路线，真诚倾听群众呼声，真实反映群众意愿，真情关心群众疾苦，急群众之所急，想群众之所想。以求真务实的作风推进各项工作，精简会议和文件，减少各类评比达标活动，把更多的时间和精力用于深入基层、研究政策、解决问题，促使各项决策和工作部署落到实处，取得实效。进一步增强忧患意识、机遇意识、责任意识、效能意识和服务意识，恪尽为民之责，善谋为民之策，多为群众办好事、办实事，多干打基础、利长远的事，真正做到权为民所用，情为民所系，利为民所谋。

　　保持清正廉洁，保障为民服务。坚持执政为民、从严治政，全面推进公务员队伍建设。要立足于关心干部、爱护干部、保护干部，坚持不懈地加强廉政建设，落实“一岗双责”，在坚决惩治腐败的同时，加强廉政文化建设，更加注重治本，更加注重预防，更加注重制度建设，形成拒腐防变教育长效机制、反腐倡廉制度体系、权力运行监控机制。进一步规范国有企业产权交易、工程招投标和政府采购等各项工作，治理商业贿赂。深入开展经济责任审计，强化对权力运行的制约和监督。加大对财政性投资的监督管理力度，严格控制公务消费。时刻牢记“两个务必”，树立正确的政绩观，始终坚持自重、自省、自警、自励，树立和增强公仆意识，真正做到为人民服务，对人民负责，受人民监督，让人民满意。

　　各位代表！让我们更加紧密地团结在以胡锦涛同志为总书记的党中央周围，高举中国特色社会主义伟大旗帜，全面落实科学发展观，加快构建社会主义和谐社会，在中共厦门市委的坚强领导下，紧紧依靠全市人民，万众一心，抢抓机遇，克服困难，推动厦门经济社会又好又快发展，为夺取全面建设小康社会新胜利、谱写厦门人民美好生活的新篇章而努力奋斗！
